# Iodine-Negative Rare Gluteal Muscle Metastasis of Papillary Thyroid Cancer: Detected by ^68^ Ga-DOTATATE PET/MRI and ^18^ F-FDG PET/CT

**DOI:** 10.1055/s-0045-1809423

**Published:** 2025-06-03

**Authors:** Seckin Bilgic, Irem Koroglu, M. Sait Sager, Kerim Sonmezoglu

**Affiliations:** 1 Department of Nuclear Medicine, Uludag Medical Faculty, Uludag University, Bursa, Türkiye; 2 Department of Nuclear Medicine, Cerrahpasa Medical Faculty, Istanbul University, Cerrahpasa, Istanbul, Türkiye

**Keywords:** papillary thyroid carcinoma, gluteal muscles, RAI-refractory, ^68^ Ga-DOTATATE PET/MRI, ^18^ F-FDG PET/CT

## Abstract

The case presents a 57-year-old male with metastatic papillary thyroid carcinoma (PTC) to the lymph nodes, lung, and mediastinum. Despite receiving multiple high-dose radioactive iodine (RAI) therapies, the patient's serum thyroglobulin levels continued to rise. The patient, who was unresponsive to RAI therapy, was being evaluated for suitability for ^177^ Lu-DOTATATE therapy. Therefore, after the third high-dose treatment, simultaneous ^68^ Ga-DOTATATE PET/MRI and ^18^ F FDG PET/CT imaging were performed, revealing a painless mass in the left gluteal region. The gluteal mass was excised, and histopathology confirmed it as metastatic PTC. Muscle metastases are extremely rare for PTC. This case exemplifies the different levels of tumoral affinity shown by aggressive variants of PTC across three distinct imaging modalities: ^68^ Ga-DOTATATE PET/MRI, ^18^ F FDG PET/CT, and whole-body iodine scintigraphy.

## Introduction

Papillary thyroid carcinoma (PTC) is the most common cancer of the thyroid gland. 1 While cervical lymph node metastasis appears more frequently, the rate of distant metastasis is low. Distant metastases frequently occur in the lungs and bones and to a lesser extent in brain and liver tissue. 2 In our case, we aimed to present a rare metastasis of PTC to the gluteal muscles. 3 4

## Case Report

A 57-year-old male patient presented with complaints of neck swelling, shortness of breath, and difficulty swallowing. During his examinations, an approximately 5 cm solid nodule in the left thyroid lobe was found. Fine-needle aspiration biopsy suggested PTC, associated with pathological lymph nodes in the left cervical lymphatic stations. Consequently, he underwent a total thyroidectomy with central neck dissection and left modified lateral neck dissection. Histopathological examination revealed diffuse sclerosing variant papillary thyroid cancer with a partial tall cell variant in the left lobe, with the largest tumor size being 5 cm.

**Fig. 1:**
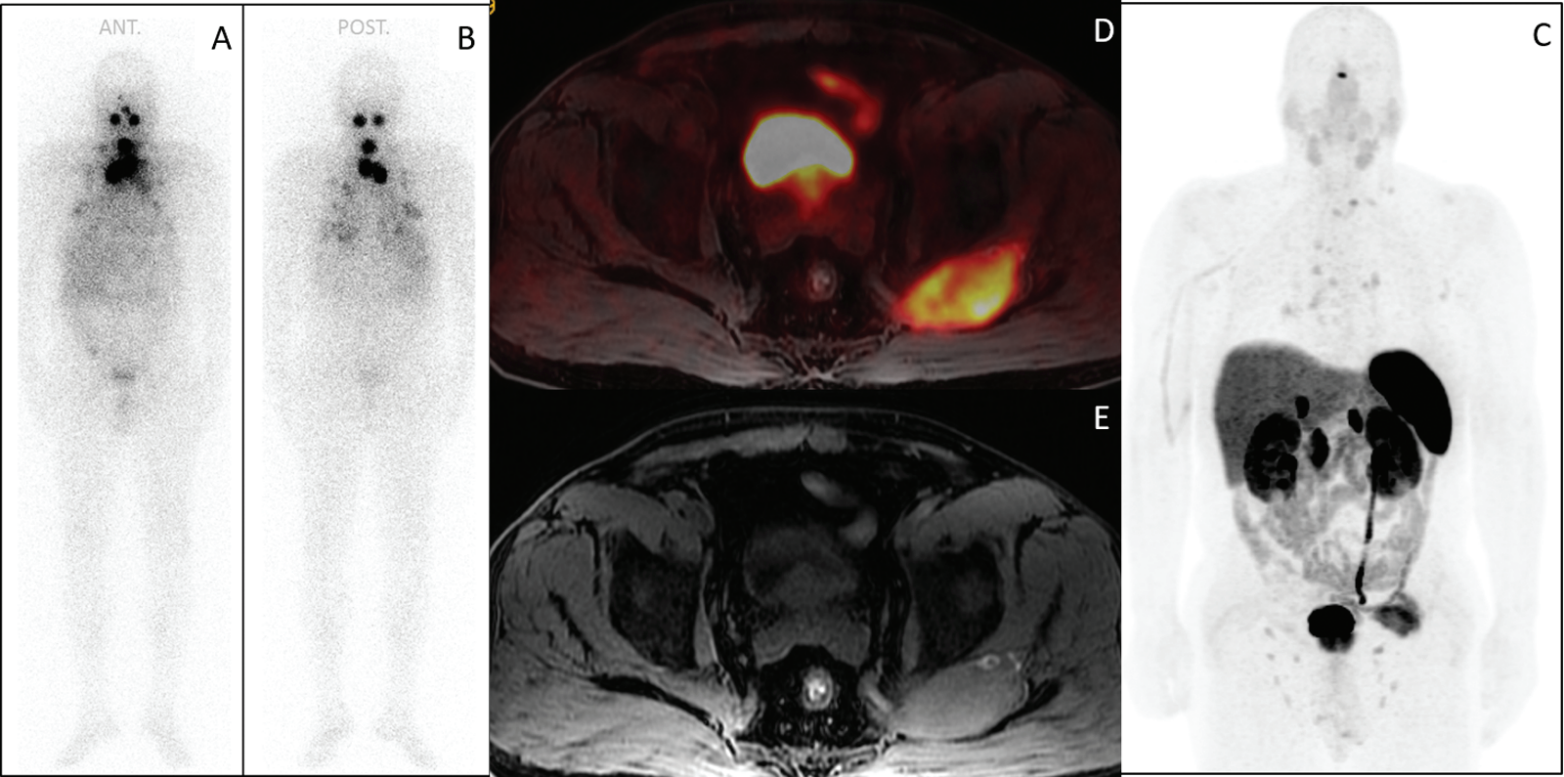
RAI-WBS and ^68^ Ga DOTATATE PET/MRI. The initial posttherapeutic 131I whole-body scan ( **A, B** ) shows three focal uptakes in the bilateral thyroid beds, mediastinal lymph nodes, and bilateral lungs. Maximum intensity projection (MIP) images of ^68^ Ga-DOTATATE PET/MRI ( **C** ) show increased uptake in cervical and mediastinal lymph nodes, lung metastases, and the left gluteal muscle. Fusion PET/MRI images ( **D, E** ) reveal a 7 cm gluteal mass with intense somatostatin receptor expression. MRI, magnetic resonance imaging; PET, positron emission tomography.

**Fig. 2:**
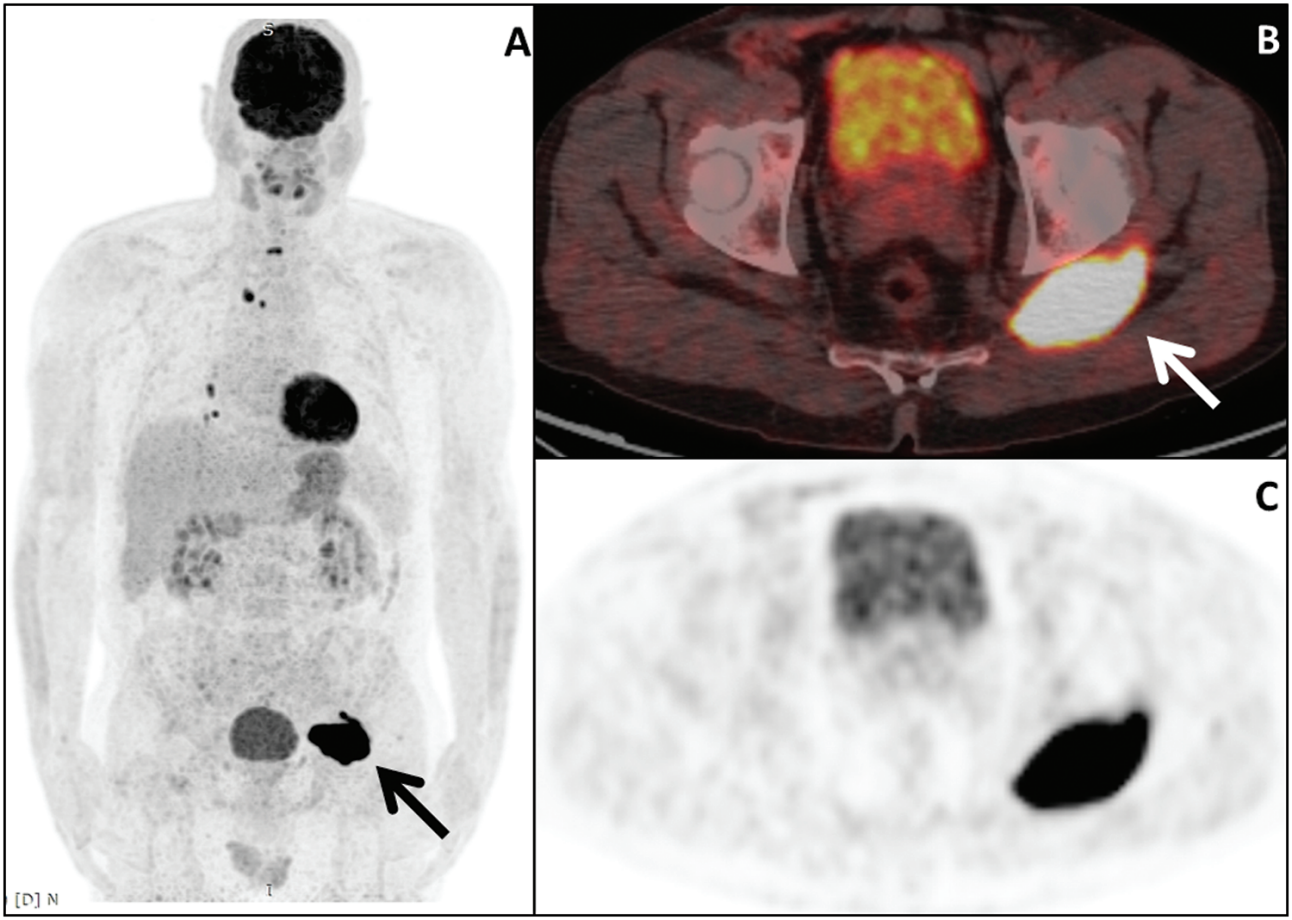
FDG PET/CT. ^18^ F-FDG PET/CT reveals hypermetabolic lesions in the mediastinum, lungs, and a mass in the left gluteal region. ( **A** ) Maximum intensity projection (MIP). ( **B, C** ) Solid arrow: axial PET/CT images demonstrate focal FDG uptake in the left gluteal muscle. CT, computed tomography; PET, positron emission tomography.

**Fig. 3:**
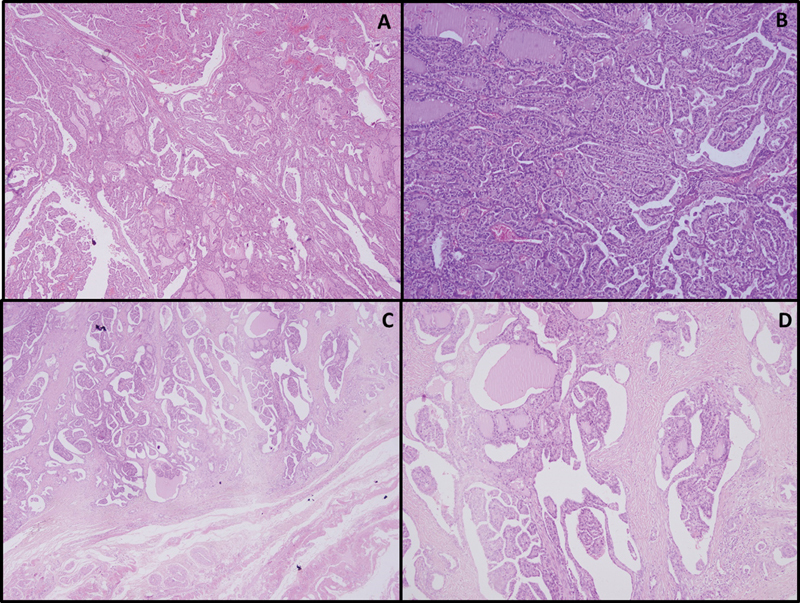
Histopathology consistent with metastatic papillary thyroid cancer. Histological examination from the total thyroidectomy revealed diffuse sclerosing and tall cell variant papillary thyroid cancer, shown in H&E stain at 40× ( **A** ) and 100× ( **B** ) magnification. ( **C, D** ) H&E-stained sections of the left gluteal mass confirm metastatic papillary thyroid carcinoma at 40× magnification.

After the surgery, serum thyroglobulin (Tg) levels were above 500 ng/mL, suggesting distant metastasis. The patient received high-dose ^131^ I therapy based on the aggressive histological subtype and radiological evidence of distant metastases, as per institutional practice. Radioiodine therapy was administered following recombinant human thyroid-stimulating hormone stimulation, in accordance with institutional protocol. Whole-body iodine scintigraphy performed after radioactive iodine (RAI) treatment showed intense increased activity of three foci in the bilateral thyroid beds and multiple masses in the mediastinum and lungs ( Fig. 1A, B ). RAI therapy with the same dose was repeated three times at 6-month intervals. A ^68^ Ga-DOTATATE PET/MRI (positron emission tomography/magnetic resonance imaging) scan revealed a 7-cm soft tissue mass adjacent to the left gluteal muscle with moderate uptake ( Fig. 1C–E ). ^18^ F-FDG PET/CT (computed tomography) images showed intense focal ^18^ F-FDG uptake in the mediastinum (SUV _max_ : 6.6), lungs (SUV _max_ : 4.5), and the left gluteal muscle (SUV _max_ : 6.2; Fig. 2A–C ). The soft tissue mass was excised. Histopathological findings were consistent with metastasis of primary PTC ( Fig. 3 ). Immunohistochemical analysis confirmed Tg, TTF-1, and PAX8 positivity in the gluteal lesion, while PSA and PSAP were negative, supporting thyroidal origin and excluding a neuroendocrine neoplasm. The gluteal lesion showed no uptake on post-therapy ^131^ I whole-body scintigraphy, despite being clearly visualized on both ^68^ Ga-DOTATATE PET/MRI and ^18^ F-FDG PET/CT. Combined with persistently elevated Tg levels, these findings fulfill the American Thyroid Association (ATA) 2015 criteria for RAI-refractory disease.

## Discussion

This case stands out by illustrating a rare gluteal muscle metastasis of PTC, confirmed histopathologically and characterized through triple-imaging correlation. While RAI-refractoriness is a well-documented phenomenon in aggressive PTC variants, gluteal muscle involvement is exceedingly uncommon and poses significant diagnostic challenges.

The gluteal lesion was not visualized on post-therapy ^131^ I whole-body scintigraphy, despite being clearly detected on both ^68^ Ga-DOTATATE PET/MRI and ^18^ F-FDG PET/CT. Persistently elevated Tg levels supported the diagnosis. According to the 2015 ATA guidelines, lack of iodine uptake in metastatic lesions with progression or biochemical activity confirms RAI-refractory disease. 5

This case also offered a unique opportunity to compare three functional imaging modalities in a single patient. Notably, all metastatic lesions were visualized on ^68^ Ga-DOTATATE PET/MRI, while both ^131^ I-WBS and ^18^ F-FDG PET/CT failed to capture all sites. Moreover, the degree of uptake varied between modalities, suggesting heterogeneity in somatostatin receptor expression and glucose metabolism. Such variability underscores the importance of complementary imaging strategies in patients with aggressive or atypical disease behavior.

PET/MRI was chosen over PET/CT for its superior soft-tissue contrast and lower radiation dose—especially relevant in a patient with prior cumulative high-dose RAI exposure. It provided both anatomical precision and functional insight, which were essential in delineating the infiltrative gluteal lesion extending from the gluteus medius toward the gluteus minimus.

Although skeletal muscle metastases from PTC have been previously described, gluteal muscle involvement remains a rare entity. 6 7 Most reports focus on FDG PET/CT, while our case demonstrates the added diagnostic value of ^68^ Ga-DOTATATE PET/MRI, particularly for anatomical clarity and lesion extent.

Almeida et al recently compared ^68^ Ga-DOTATATE PET/CT and ^18^ F-FDG PET/CT in patients with TENIS (thyroglobulin-elevated negative iodine scintigraphy) syndrome under both suppressed and stimulated thyroid-stimulating hormone (TSH) conditions. They found DOTATATE PET/CT detected more locoregional and distant lymph node metastases regardless of TSH level, although stimulation led to decreased specificity. 8 In our case, DOTATATE PET/MRI performed under TSH suppression still demonstrated clinically significant uptake, reinforcing its potential value even in nonstimulated settings.

Furthermore, Binse et al demonstrated that ^68^ Ga-DOTATOC PET/CT identified tumor lesions in 33% of patients with elevated Tg despite negative RAI and FDG scans—primarily in poorly differentiated carcinomas. 9 Only one of five papillary carcinoma patients in their cohort had a positive scan. In contrast, our case represents a rare example of ^68^ Ga-DOTATATE PET/MRI successfully detecting all metastatic sites in an aggressive variant of PTC, emphasizing the evolving role of somatostatin receptor imaging even in selected papillary subtypes.

## Conclusion

Beyond the well-known phenomenon of iodine-negativity in aggressive PTC, this case contributes an uncommon anatomical presentation and emphasizes the role of multimodal imaging in the early identification of extra-axial muscle metastases.
